# Prognostic Value of Systemic Inflammation Markers and Clinical Factors in Grade 4 Diffuse Glioma: A Multicenter Retrospective Real-World Study

**DOI:** 10.3390/cancers18193192

**Published:** 2026-10-02

**Authors:** Ece Ulukal Karancı, Gamze Emin, Ahmet Oruç, Esma Uğuztemur, Atila Yıldırım, Melek Karakurt Eryılmaz, Asım Armağan Aydın

**Affiliations:** 1 Department of Medical Oncology, Antalya City Hospital, Antalya 07080, Türkiye; 2 Department of Medical Oncology, Faculty of Medicine, Karadeniz Technical University, Trabzon 61080, Türkiye; gamzeemin@ktu.edu.tr (G.E.); atilayildirim@ktu.edu.tr (A.Y.); 3 Department of Medical Oncology, Meram Faculty of Medicine, Necmettin Erbakan University, Konya 42080, Türkiye; ahmet.oruc@erbakan.edu.tr (A.O.); mkeryilmaz@erbakan.edu.tr (M.K.E.); 4 Department of Medical Oncology, Adıyaman Training and Research Hospital, Adıyaman 02040, Türkiye; esma.uguztemur@saglik.gov.tr; 5 Department of Medical Oncology, Antalya Training and Research Hospital, Antalya 07100, Türkiye; asimarmagan.aydin@saglik.gov.tr

**Keywords:** glioblastoma, grade 4 diffuse glioma, systemic inflammation response index, hemoglobin, smoking, prognosis, survival, machine learning

## Abstract

**Background/Objectives:** Systemic inflammation contributes to tumor growth and spread. We aimed to evaluate the association of pretreatment inflammatory indices and clinical and laboratory factors with survival in newly diagnosed grade 4 diffuse glioma. **Methods:** A total of 120 patients diagnosed with grade 4 diffuse glioma at four centers between 2013 and 2026 were analyzed retrospectively. Nine indices derived from complete blood counts were calculated. Overall survival (OS) and progression-free survival (PFS) were analyzed with Kaplan–Meier and multivariable Cox regression. The cut-off for the systemic inflammation response index (SIRI) was determined with maximally selected rank statistics. Ten machine learning models were compared with the Cox model using repeated cross-validation. **Results:** Median follow-up was 70 months. Median OS was 22.8 months (95% confidence interval [CI] 15.8–26.3) and median PFS was 10.9 months (95% CI 9.1–14.4). In the multivariable model, SIRI > 2.85 was independently associated with OS (hazard ratio [HR] 2.62, 95% CI 1.38–4.96, *p* = 0.003), and this association persisted in the isocitrate dehydrogenase (IDH)-wildtype subgroup (HR 3.06, 95% CI 1.27–7.39, *p* = 0.013). Hemoglobin was independently associated with PFS (HR per g/dL 0.84, 95% CI 0.73–0.96, *p* = 0.011). Smoking was an independent indicator of poor prognosis for both OS (HR 2.02) and PFS (HR 1.73). Ki-67 and tumor size were not associated with survival. Machine learning models did not outperform the Cox model, and their variable importance rankings were consistent with the classical analysis. **Conclusions:** Pretreatment SIRI, hemoglobin, and smoking were independently associated with survival in grade 4 diffuse glioma. These markers are available from routine tests and may contribute to prognostic assessment.

## Simple Summary

Glioblastoma and other grade 4 diffuse gliomas are the most aggressive brain tumors in adults. Clinicians still have few simple tools to estimate prognosis for individual patients. In this study of 120 patients treated at four centers, we examined whether routine blood tests and basic clinical information can help. Patients with a high systemic inflammation response index, a value calculated from a routine blood count, had shorter survival. Patients with lower hemoglobin experienced earlier disease progression. Smoking was associated with worse outcomes for both survival and progression. In contrast, tumor size and the Ki-67 proliferation marker did not predict survival. Machine learning models confirmed the importance of the same factors. These results suggest that inexpensive information already available in routine practice may help clinicians estimate prognosis in grade 4 diffuse glioma.

## 1. Introduction

Glioblastoma is the most common malignant primary brain tumor in adults [1]. Standard treatment consists of surgical resection, radiotherapy, and temozolomide. Despite this treatment, median survival usually remains below two years [2,3]. The 2021 World Health Organization classification separated isocitrate dehydrogenase (IDH)-mutant grade 4 astrocytomas from glioblastoma [4]. In daily practice, however, both groups are managed with similar treatment approaches.

Systemic inflammation is one of the processes that support tumor growth and spread [5,6,7]. Indices derived from complete blood counts reflect this response in an inexpensive and accessible way [8]. The prognostic value of these markers, most notably the neutrophil-to-lymphocyte ratio (NLR), has been shown in many solid tumors [9]. The systemic inflammation response index (SIRI), which combines neutrophil, monocyte, and lymphocyte counts in a single formula, was first described in pancreatic cancer [10]. Associations between SIRI and similar indices and survival have also been reported in glioma cohorts [11,12,13]. However, most of these studies are single-center, and reported cut-off values differ considerably. Data on the prognostic effect of factors such as smoking and anemia are limited, and conflicting results have been reported [14,15].

In this multicenter retrospective study, we evaluated the association of pretreatment inflammatory indices and clinical factors with overall survival (OS) and progression-free survival (PFS) in patients with newly diagnosed grade 4 diffuse glioma. The contribution of this study lies in the combination of several features. Nine complete blood count-derived indices were evaluated simultaneously in a multicenter real-world cohort with a long follow-up. Smoking and hemoglobin were examined together with these indices. The classical Cox model was compared with ten machine learning models. The main analysis was also repeated in the IDH-wildtype subgroup.

## 2. Materials and Methods

### 2.1. Study Design and Patients

This retrospective cohort study was conducted with the participation of the medical oncology clinics of four centers. The study was approved by the Scientific Research Ethics Committee of Antalya Training and Research Hospital (date: 8 January 2026; decision no: 1/11; protocol code: 2026-11). Patients aged over 18 years with histologically confirmed grade 4 diffuse glioma (glioblastoma, IDH-wildtype, or astrocytoma, IDH-mutant, grade 4) diagnosed between January 2013 and January 2026 were included. After removal of one duplicate record, analyses were performed on 120 patients.

### 2.2. Data Collection and Definitions

Demographic characteristics, comorbidity, smoking status, Eastern Cooperative Oncology Group (ECOG) performance status [16], presenting symptom, tumor location and size, type of surgery, pathological diagnosis, IDH status, Ki-67 index, and oncological treatments were recorded from patient files. Pretreatment complete blood count and biochemistry values were collected. Blood values were obtained at the diagnostic admission of each patient, before surgery and before the start of any oncological treatment. Leukocyte subsets and platelet counts were converted to a common unit (109/L) across centers. C-reactive protein (CRP) values were converted to mg/L based on the units reported by each center. Platelet morphology parameters such as mean platelet volume (MPV) and platelet distribution width (PDW) were not included in the analysis because a device-related scale difference was detected between centers.

Inflammatory and nutritional indices were calculated as follows: NLR = neutrophils/lymphocytes; derived NLR (dNLR) = neutrophils/(leukocytes − neutrophils); platelet-to-lymphocyte ratio (PLR) = platelets/lymphocytes; monocyte-to-lymphocyte ratio (MLR) = monocytes/lymphocytes; systemic immune-inflammation index (SII) = platelets × neutrophils/lymphocytes [17]; SIRI = neutrophils × monocytes/lymphocytes [10]; pan-immune-inflammation value (PIV) = platelets × neutrophils × monocytes/lymphocytes [18]; prognostic nutritional index (PNI) = 10 × albumin (g/dL) + 5 × lymphocytes [19]; CRP-to-albumin ratio (CAR) = CRP/albumin.

OS was defined as the time from diagnosis to death. For surviving patients, follow-up was limited to the date of the last visit. PFS was defined as the time from diagnosis to radiological or clinical progression or death. One patient with progression but an unknown progression date was excluded from the PFS analysis.

### 2.3. Statistical Analysis

Continuous variables were presented as median (interquartile range, IQR) and categorical variables as number (percentage). Survival was analyzed with the Kaplan–Meier method, and groups were compared with the log-rank test. Follow-up time was calculated with the reverse Kaplan–Meier method. The cut-off value for SIRI was determined on OS using maximally selected rank statistics [20]. In this procedure, all unique SIRI values within the 10th to 90th percentiles of the distribution were evaluated as candidate cut-off points, with a minimal group proportion of 0.10. In our data, 96 candidate values between 0.67 and 6.02 were assessed, and the value with the largest standardized log-rank statistic was selected. Because the cut-off of 2.85 was derived from the data, it should be considered exploratory rather than a validated threshold. The reliability of this cut-off was tested with additional analyses. SIRI was also modeled after a median split and as a continuous variable. In addition, the adjusted hazard ratio for SIRI was recalculated for a grid of alternative cut-off values from the 15th to the 85th percentile of its distribution in 5-percentile steps.

Age, ECOG performance status, extent of resection, and IDH status were included in the multivariable Cox regression models as prespecified clinical covariates. Smoking, which was significant in univariable analysis, was added to the model. SIRI and hemoglobin were kept together in both the OS and PFS models. Models were stratified by center to account for between-center differences. The proportional hazards assumption was tested with Schoenfeld residuals. Results of the Cox models were reported as hazard ratios (HR) with 95% confidence intervals (CI). *p* < 0.05 was considered statistically significant. All analyses were performed with R 4.5.1 (R Foundation for Statistical Computing, Vienna, Austria) [21] using the survival, survminer, maxstat, and gtsummary packages. As an additional sensitivity analysis, the multivariable models were repeated in the subgroup of patients with known IDH status.

### 2.4. Machine Learning Analysis

As an additional analysis, ten machine learning methods widely used for survival prediction were compared with the classical Cox model: least absolute shrinkage and selection operator (LASSO)-Cox, ridge-Cox, elastic net-Cox, random survival forest [22], gradient boosting (Cox loss), XGBoost-Cox, XGBoost-AFT (accelerated failure time), model-based boosting (CoxPH loss), survival support vector machine, and survival tree. Models were trained with 26 baseline clinical and laboratory variables. Performance was evaluated with Harrell’s C-index [23] using 5-fold cross-validation with 20 repeats. Missing values were imputed with the median or mode within each cross-validation fold using the training set only. The lambda parameter for the LASSO, ridge, and elastic net models was selected with inner cross-validation. Variable importance rankings were obtained from models fitted on the full dataset. These analyses used the glmnet, randomForestSRC, gbm, xgboost, mboost, survivalsvm, and rpart packages.

## 3. Results

### 3.1. Patient and Treatment Characteristics

**Figure 1:**
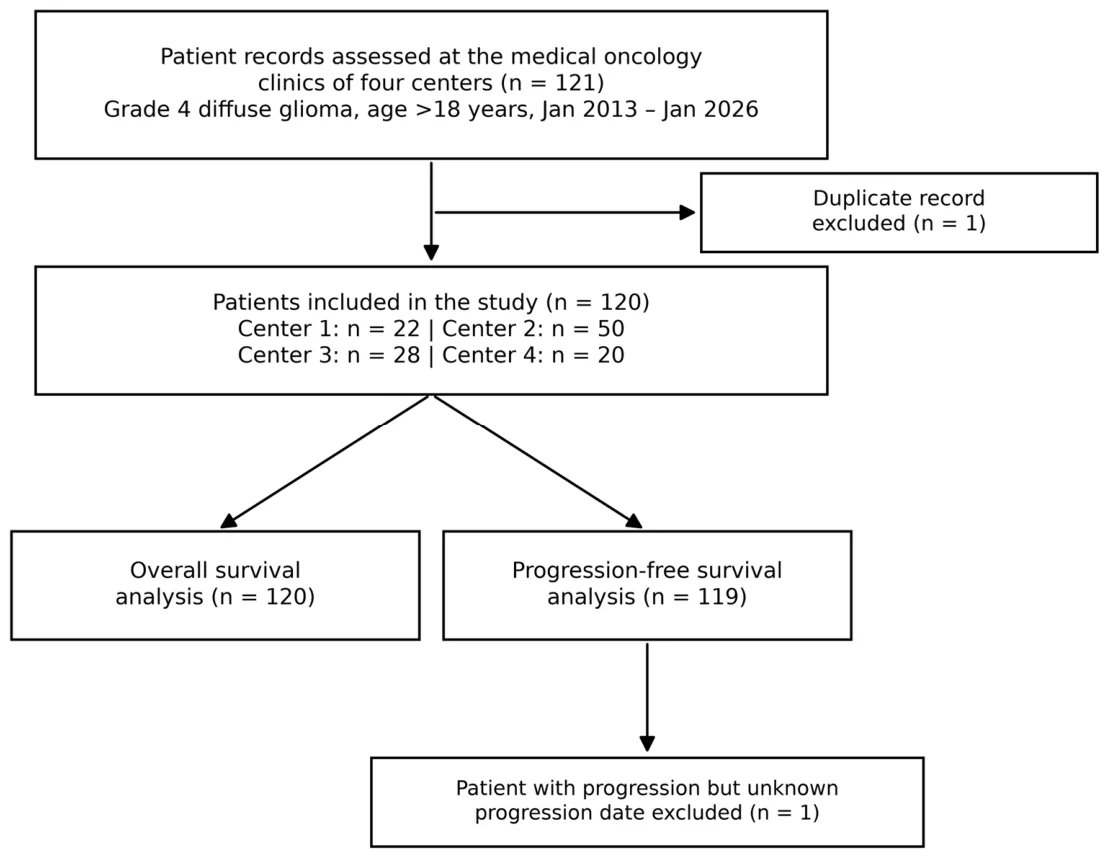
Patient flow diagram.

Of 121 records evaluated from four centers, one duplicate record was removed and 120 patients were included in the study (Figure 1). Median age was 55.5 years (IQR 49.0–64.5), and 74 patients (62%) were male. Ninety-nine patients (83%) had an ECOG performance status of 0–1. The most common presenting symptom was headache (59%). Forty-one patients (34%) were active smokers. IDH status was wildtype in 71 patients (59%) and mutant in 13 (11%). IDH status was unknown in 36 patients (30%). Gross total resection was performed in 79 patients (66%). Adjuvant radiotherapy was given to 115 patients (96%) and adjuvant chemotherapy to 118 (98%). Patient characteristics are presented in Table 1.

### 3.2. Survival

Median follow-up was 70.0 months. Death occurred in 85 patients (71%) during follow-up. Median OS was 22.8 months (95% CI 15.8–26.3), and the one- and two-year OS rates were 66.9% and 49.9%. Of the 119 patients included in the PFS analysis, 103 (87%) experienced progression or death. Median PFS was 10.9 months (95% CI 9.1–14.4).

### 3.3. Prognostic Factors

In univariable analyses, age and smoking were associated with both OS and PFS (Table 2). Hemoglobin was associated with PFS (HR per standard deviation [SD] 0.78, 95% CI 0.64–0.95, *p* = 0.017). When analyzed as continuous variables, the inflammatory indices showed no significant association with OS or PFS.

**Figure 2:**
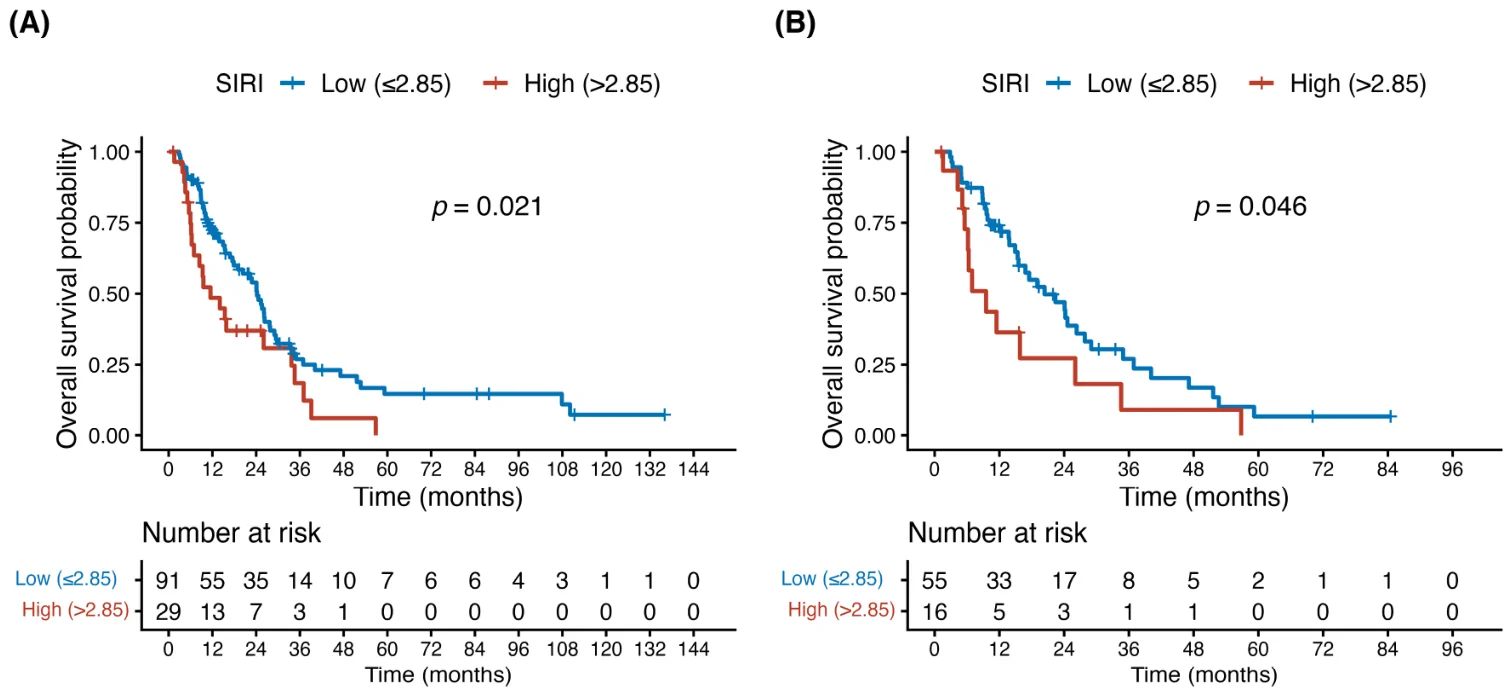
Kaplan–Meier curves for overall survival according to SIRI: (**A**) whole cohort; (**B**) IDH-wildtype subgroup. SIRI = systemic inflammation response index; IDH = isocitrate dehydrogenase.

The cut-off value determined for SIRI was 2.85. Median OS was 11.5 months in the 29 patients (24%) with SIRI > 2.85 and 24.2 months in those with SIRI ≤ 2.85 (log-rank *p* = 0.021; Figure 2A). The same difference was observed in the IDH-wildtype subgroup (9.5 versus 20.4 months; log-rank *p* = 0.046; Figure 2B).

**Figure 3:**
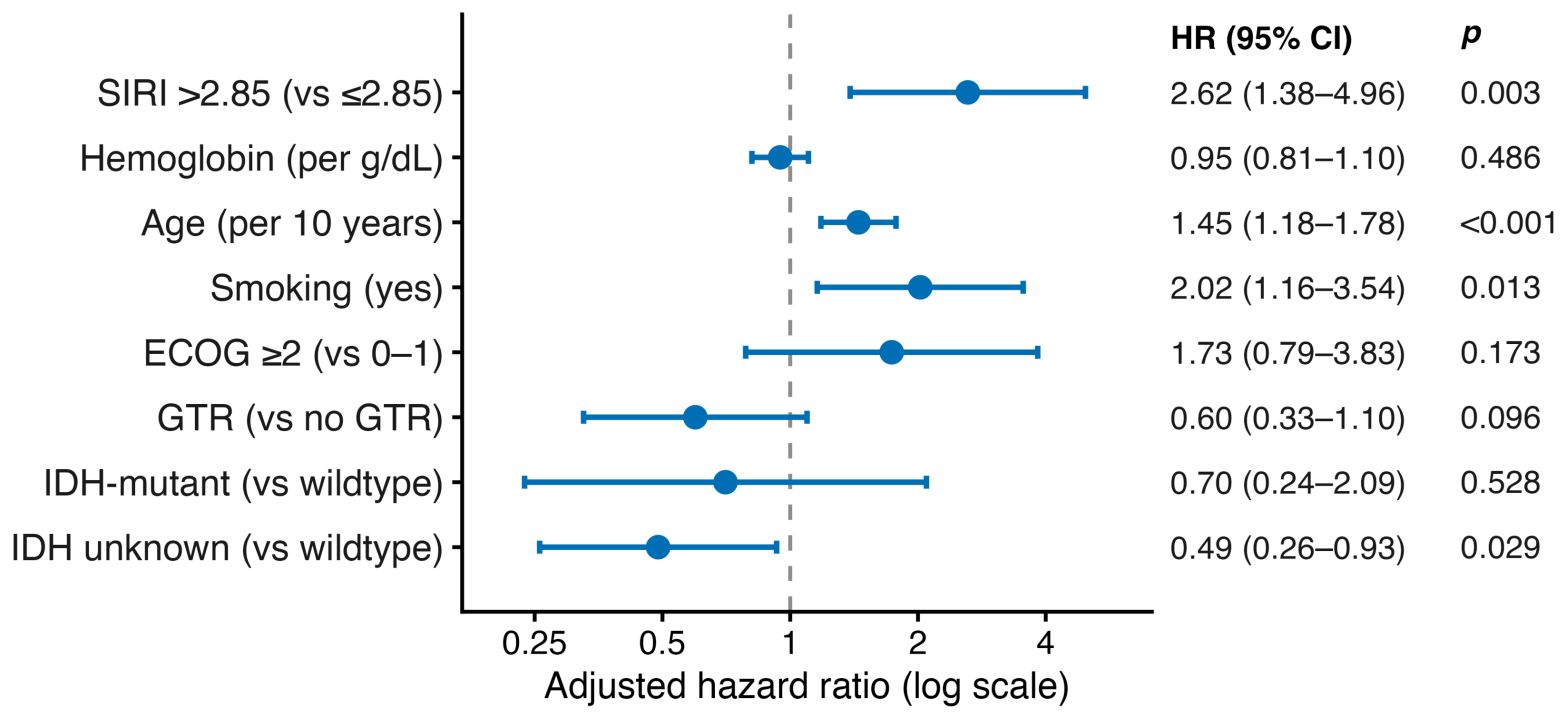
Forest plot of the multivariable Cox model for overall survival (stratified by center). The dashed vertical line indicates a hazard ratio of 1. HR = hazard ratio; CI = confidence interval; SIRI = systemic inflammation response index; ECOG = Eastern Cooperative Oncology Group; GTR = gross total resection; IDH = isocitrate dehydrogenase.

In the multivariable model, SIRI >2.85 was an independent indicator of poor prognosis for OS (HR 2.62, 95% CI 1.38–4.96, *p* = 0.003) (Table 3, Figure 3). Age (HR per 10 years 1.45, 95% CI 1.18–1.78, *p* < 0.001) and smoking (HR 2.02, 95% CI 1.16–3.54, *p* = 0.013) were the other variables independently associated with OS. The association between SIRI and OS was more pronounced in the model restricted to IDH-wildtype patients (HR 3.06, 95% CI 1.27–7.39, *p* = 0.013) (Supplementary Table S1). Across a grid of alternative cut-off values from the 15th to the 85th percentile in 5-percentile steps, the adjusted association with OS was present for cut-offs between 2.65 and 3.67 (approximately the 75th to 85th percentiles) and was not observed for lower cut-off values (Supplementary Figure S1 and Supplementary Table S2). When SIRI was modeled as a continuous variable, the adjusted HR per one standard deviation was 1.17 (95% CI 0.92–1.48, *p* = 0.20). With a median split (1.58), the adjusted HR was 1.11 (95% CI 0.68–1.83, *p* = 0.67).

**Figure 4:**
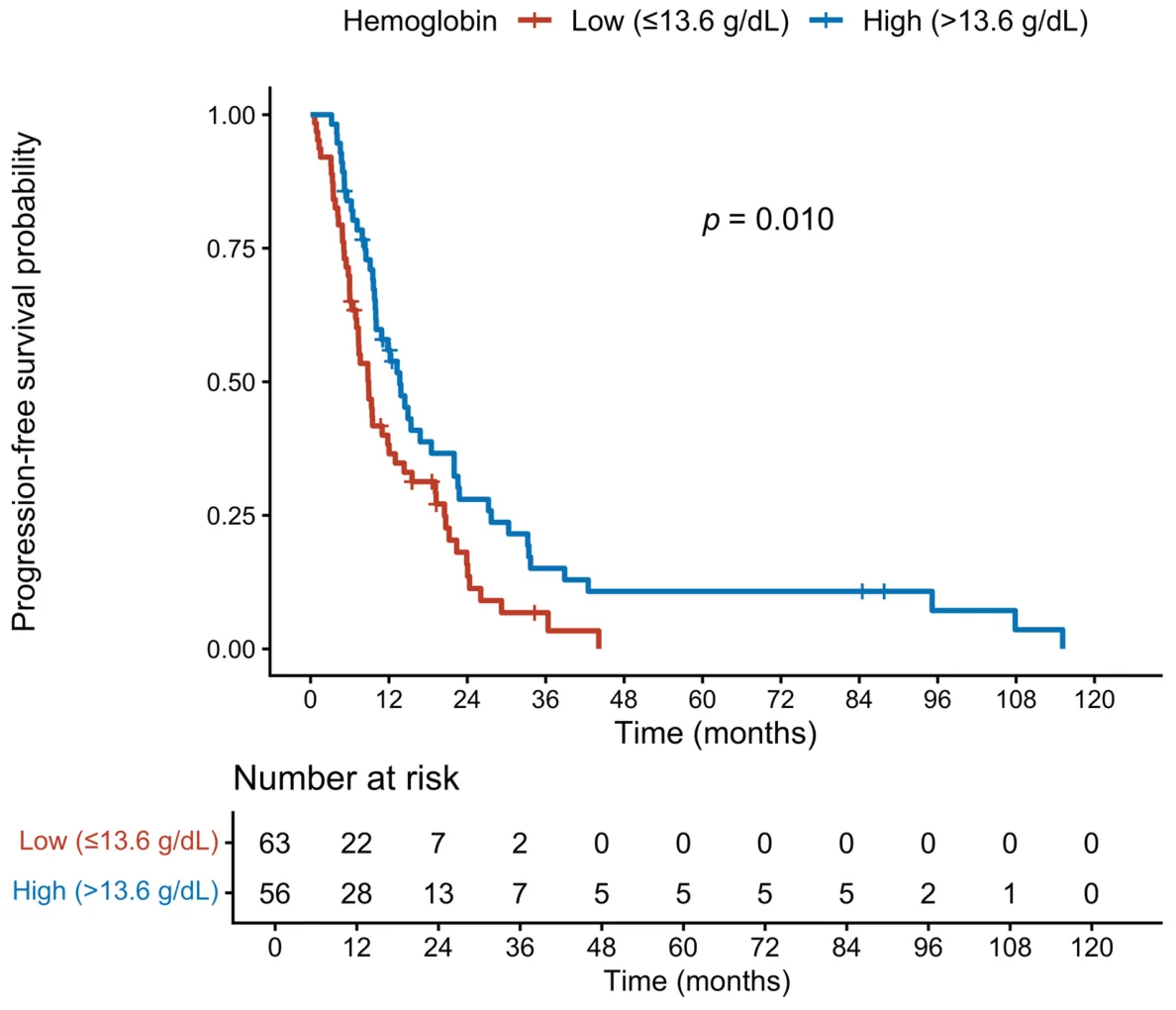
Kaplan–Meier curves for progression-free survival according to the median hemoglobin value (13.6 g/dL).

In the multivariable PFS model, hemoglobin was an independent prognostic factor (HR per g/dL 0.84, 95% CI 0.73–0.96, *p* = 0.011). Median PFS was 8.9 months in patients with hemoglobin below the median (13.6 g/dL) and 13.7 months in those above it (Figure 4). Smoking (HR 1.73, 95% CI 1.06–2.81, *p* = 0.029), age (HR per 10 years 1.25, *p* = 0.015), and gross total resection (HR 0.49, 95% CI 0.29–0.83, *p* = 0.008) were independently associated with PFS. SIRI was not associated with PFS (HR 1.24, *p* = 0.48). Hemoglobin was not significant in the OS model (HR 0.95, *p* = 0.49). The proportional hazards assumption held in all models. In the sensitivity analysis restricted to the 84 patients with known IDH status, the direction and size of the SIRI association were preserved (HR 2.02, 95% CI 0.87–4.66), but the association did not reach statistical significance, in keeping with the smaller number of events. Smoking, age, and gross total resection remained significant in this subgroup (Supplementary Table S3).

Ki-67 and tumor size were not associated with survival. This result was the same in continuous analyses and in groups formed with cut-off values. None of the other inflammatory indices showed an independent association in the multivariable models.

### 3.4. Machine Learning Models

In repeated cross-validation, none of the ten machine learning models achieved a better C-index than the classical Cox model (Supplementary Figure S2 and Supplementary Table S4). The variable importance rankings of the models were largely consistent with the classical analysis. Age was the most important variable in all models. Smoking, hemoglobin, and inflammation-related variables (monocytes, MLR, SIRI) were ranked highly across algorithms.

## 4. Discussion

In this study, we examined 120 patients with newly diagnosed grade 4 diffuse glioma from four centers. Three main findings emerged. First, OS was independently shorter in patients with a pretreatment SIRI above 2.85. This association was more pronounced in the IDH-wildtype subgroup. Second, low hemoglobin was independently associated with PFS only. Third, smoking was an independent indicator of poor prognosis for both OS and PFS. In contrast, tumor-related markers such as Ki-67 and tumor size were not associated with survival. This pattern suggests that part of the prognostic information comes from the systemic state of the patient rather than from the tumor itself.

In our study, median OS was 11.5 months in patients with SIRI above 2.85 and 24.2 months in those below. The difference persisted in the multivariable model (HR 2.62). The literature points in the same direction. In a meta-analysis of 2275 glioma patients by Lei et al., high NLR was associated with worse OS (HR 1.43) [11]. In a meta-analysis of 10 studies and 1942 patients, Jiang and Zhou reported that high SIRI was associated with poor prognosis for both OS (HR 1.67) and PFS (HR 1.80) [12]. In 291 glioblastoma patients who underwent gross total resection, Wang et al. found high preoperative SIRI associated with shorter survival after propensity score matching [13]. Topkan et al. found median values very close to ours (12.2 versus 22.9 months) for SIRI groups in 181 glioblastoma patients treated with the Stupp protocol [24]. Our results are in line with these studies. A possible biological explanation has been described in the literature [5,6,7,8]. In cancer-related inflammation, neutrophils can support angiogenesis and tumor growth. Circulating monocytes can differentiate into tumor-associated macrophages in tumor tissue. Lymphopenia may reflect a weak antitumor immune response. SIRI combines these three cell counts in a single formula [10]. However, SIRI is an indirect index obtained from peripheral blood. It is not a direct measure of the tumor immune microenvironment, and blood counts can also be affected by factors such as corticosteroids, infection, seizures, and comorbidity. Our study did not include tumor tissue data such as immunohistochemistry or sequencing. We therefore interpret the SIRI finding as hypothesis-generating rather than as direct evidence of glioma-associated immune biology. Within these limits, it is still notable that NLR and SII, which do not include monocytes, did not remain independent in our study, and that monocyte-related variables ranked highly in the machine learning analyses. This observation points to the monocyte component of the index as a topic for further study rather than a proven mechanism. The stronger SIRI association in the IDH-wildtype subgroup may be explained by a clearer signal in a biologically more homogeneous group.

Two points about the SIRI finding still require attention. First, our cut-off value (2.85) is in the upper part of the range reported in the literature (0.67–3.03). Cut-off values differ considerably between studies. We therefore presented the cut-off dependence transparently, together with median split and continuous variable analyses. The value of 2.85 was derived from this dataset and has not been validated externally. It is exploratory and should not be used as a clinical decision threshold. The detailed sensitivity analysis across candidate cut-off values is presented in Supplementary Figure S1 and Supplementary Table S2. Second, corticosteroids increase neutrophil counts and decrease lymphocyte counts. They can therefore directly affect index values. Grossman et al. showed that severe CD4 lymphopenia developed in about 40% of patients with high-grade glioma receiving radiotherapy and temozolomide by the second month of treatment [25]. Because steroid doses were not recorded, we could not separate this effect.

In our study, both OS and PFS were independently shorter in smokers (HR 2.02 and 1.73, respectively). Data on this topic are limited. Smoking has mostly been studied as a risk factor for developing glioma. On the prognostic side, Carr et al. found no significant association between smoking and survival in their study of comorbidities in glioblastoma patients [14]. The difference may be explained by sample characteristics, the definition of smoking, and model differences. Continued smoking after a cancer diagnosis has been associated with worse treatment outcomes in other tumor types [26]. Our finding can be interpreted as follows: smoking is a known source of chronic systemic inflammation and damages vascular structure. It may also affect the response to radiotherapy by reducing tissue oxygenation. Patients who smoke may have more comorbid disease, and treatment adherence may be lower. Through these pathways, smoking may also affect survival indirectly. The fact that smoking and SIRI remained independent in the same model shows that the prognostic information they carry does not overlap. Still, smoking information was based on patient self-report, and pack-year data were not available. It is also difficult to separate the effect of tobacco exposure itself from systemic illness, treatment tolerance, and socioeconomic or behavioral factors. This finding should therefore be interpreted with caution.

In our study, low hemoglobin was independently associated with PFS only (HR per g/dL 0.84). Median PFS was about five months shorter in patients with hemoglobin below the median. No similar association was seen for OS. Anemia is a common finding in cancer patients, and a quantitative review across tumor types associated it with shorter survival [27]. In glioblastoma, however, the literature is inconsistent. Odrazka et al. reported longer survival in glioblastoma patients with a pre-radiotherapy hemoglobin above 12 g/dL [15]. Kaisman-Elbaz et al. found low hemoglobin associated with poor prognosis in multivariable analysis (HR 1.79) and proposed that anemia may increase invasiveness through tumor hypoxia [28]. In contrast, Pleskacova et al. found no association between hemoglobin and survival in their series of 136 patients [29]. Our finding can be interpreted as follows: anemia is a common consequence of chronic inflammation and disease burden. It may deepen hypoxia in tumor tissue. Hypoxic tumor cells are more resistant to radiotherapy and some chemotherapeutic agents [28]. The observation of the association for PFS rather than OS would be compatible with this mechanism, although other explanations are possible. The effect of anemia may be evident in the early period up to progression. Because the post-progression period is added to OS, this effect may not be reflected in total survival. Grouping according to the World Health Organization definition of anemia (<13 g/dL in men, <12 g/dL in women) was not significant, whereas continuous hemoglobin was. This indicates that the risk increases gradually as hemoglobin falls rather than at a specific threshold. Low hemoglobin may also reflect nutritional status, renal function, chronic disease, or baseline functional status rather than tumor hypoxia alone. The association with PFS but not OS should therefore not be read as evidence of a single direct mechanism.

In our study, age was independently prognostic for both endpoints and gross total resection for PFS. The prognostic value of these two factors is well known [2,30]. Gross total resection was also associated with OS in the IDH-wildtype subgroup (Supplementary Table S1). The presence of these expected associations in our models supports the internal consistency of the dataset. The lack of significance for Ki-67 and tumor size is an expected result. These markers are more useful for separating low-grade from high-grade tumors. Because our cohort consisted entirely of grade 4 tumors, their discriminative power remained limited.

In the machine learning analyses, different algorithms brought age, smoking, hemoglobin, and inflammation variables to the top of the variable importance rankings. In contrast, no model achieved a better C-index than the classical Cox model. Penalized Cox models (LASSO, ridge, elastic net) showed the performance closest to the classical model. Tree-based models and the survival support vector machine remained behind. A systematic review by Christodoulou et al. showed that machine learning provides no average benefit over regression for clinical prediction models [31]. This result can be explained by sample size. For complex models to gain an advantage, enough patients are needed to learn nonlinear relationships between variables. A cohort of 120 patients is not sufficient for this. The consistency of the importance rankings shows that our main findings do not depend on the modeling method.

The strengths of our study include its multicenter real-world data, a median follow-up of 70 months, and careful standardization of laboratory units across centers. Analyses were performed with center-stratified models, and cut-off dependence was presented transparently with sensitivity analyses. Evaluating OS and PFS together allowed us to see the endpoint-specific behavior of the markers. The reproduction of the findings with different modeling approaches also increases the reliability of the results.

Our study has several limitations. The retrospective design led to missing data that depended on record quality. The sample is relatively small, and confidence intervals are wide, particularly in the subgroup analyses. The cohort consists of patients who reached oncology clinics, most of whom were able to receive adjuvant treatment. This may have introduced selection bias. The long accrual period may also include changes in clinical practice over time. Laboratory values were measured at a single time point, and their changes over time were not evaluated. Acute conditions such as infection at the time of measurement were not recorded and may have affected the values. Corticosteroid doses were also not recorded. O6-methylguanine-DNA methyltransferase (MGMT) methylation status could not be included because it was unknown in most patients. IDH status was unknown in 30% of patients. This mainly reflects earlier diagnostic years, in which IDH testing was not routine. CRP and body mass index data were missing in some patients. Progression assessment was based on the radiological practice of each center, and no central image review according to standardized criteria such as Response Assessment in Neuro-Oncology (RANO) was performed [32]. The SIRI cut-off value was derived from this dataset. It therefore requires validation in other cohorts. For the machine learning models, no extensive hyperparameter search was performed, and commonly used standard values were preferred. Finally, platelet morphology parameters could not be evaluated because of device differences. Smoking status was based on self-report, and pack-year data were not available. Because of the missing molecular data, we could not fully determine whether the associations of SIRI and hemoglobin with survival are independent of established molecular prognostic factors such as MGMT methylation.

## 5. Conclusions

In this multicenter retrospective study, high pretreatment SIRI was independently associated with OS and low hemoglobin with PFS in grade 4 diffuse glioma. Smoking was an indicator of poor prognosis for both endpoints. These markers can be obtained from routine tests and may provide a low-cost contribution to prognostic assessment. The findings need support from prospective and externally validated studies.

## Figures and Tables

**Table 1:** Baseline characteristics of the study population (N = 120).

	All Patients (N = 120) ^1^
Age, years	55.5 (49.0–64.5)
Sex	
Male	74 (62%)
Female	46 (38%)
Comorbidity	48 (41%)
Missing	2
Smoking	41 (34%)
History of prior brain tumor	6 (5.0%)
ECOG performance status	
0–1	99 (83%)
≥2	21 (18%)
Presenting symptom	
Headache	71 (59%)
Seizure or syncope	28 (23%)
Visual disturbance	2 (1.7%)
Motor deficit	9 (7.5%)
Numbness	4 (3.3%)
Other	6 (5.0%)
Neurological deficit at diagnosis	27 (23%)
Epileptic seizure at diagnosis	27 (23%)
Histological diagnosis	
Astrocytoma, IDH-mutant, grade 4	16 (16%)
Glioblastoma, IDH-wildtype	86 (84%)
Missing	18
IDH status	
IDH-wildtype	71 (59%)
IDH-mutant	13 (11%)
Unknown	36 (30%)
Laterality	
Left	60 (50%)
Right	59 (50%)
Missing	1
Tumor location	
Frontal	50 (42%)
Parietal	25 (21%)
Temporal	25 (21%)
Occipital	4 (3.3%)
Multiple lobes	14 (12%)
Deep or midline	2 (1.7%)
Focality	
Unifocal	112 (93%)
Multifocal	8 (6.7%)
Tumor size, mm	43.0 (35.0–50.0)
Missing	6
Ki-67, %	20.0 (15.0–40.0)
Missing	39
Extent of resection	
Biopsy	7 (5.8%)
Subtotal resection	34 (28%)
Gross total resection	79 (66%)
Adjuvant radiotherapy	114 (95%)
Radiotherapy dose, Gy	
30	1 (1.1%)
34	1 (1.1%)
36	1 (1.1%)
40	2 (2.2%)
56	1 (1.1%)
60	83 (93%)
Missing	31
Adjuvant chemotherapy	117 (98%)
Response to adjuvant therapy	
Complete response	20 (17%)
Residual disease under follow-up	25 (21%)
Not completed or progression during therapy	72 (62%)
Missing	3
White blood cells, ×10^9^/L	8.2 (6.7–10.3)
Neutrophils, ×10^9^/L	5.4 (4.0–7.2)
Lymphocytes, ×10^9^/L	1.7 (1.3–2.4)
Monocytes, ×10^9^/L	0.6 (0.5–0.7)
Platelets, ×10^9^/L	242.5 (191.0–294.0)
Hemoglobin, g/dL	13.6 (12.2–14.7)
Albumin, g/dL	3.9 (3.6–4.2)
Missing	2
C-reactive protein, mg/L	7.0 (3.2–17.1)
Missing	44
Lactate dehydrogenase, U/L	222.0 (185.0–274.0)
Missing	19
NLR	3.0 (2.0–4.7)
dNLR	2.0 (1.4–2.9)
PLR	141.8 (100.8–217.9)
MLR	0.32 (0.23–0.48)
SII	708.3 (455.1–1186.6)
SIRI	1.58 (1.09–2.66)
PIV	373.6 (244.8–735.9)
PNI	47.6 (44.1–52.1)
Missing	2
CAR	1.85 (0.76–4.30)
Missing	45
Progression or recurrence	96 (80%)
Death during follow-up	85 (71%)

^1^ Data are n (%) or median (IQR). Abbreviations: CAR = C-Reactive Protein-to-Albumin Ratio; dNLR = Derived Neutrophil-to-Lymphocyte Ratio; ECOG = Eastern Cooperative Oncology Group; IDH = Isocitrate Dehydrogenase; IQR = Interquartile Range; MLR = Monocyte-to-Lymphocyte Ratio; NLR = Neutrophil-to-Lymphocyte Ratio; PIV = Pan-Immune-Inflammation Value; PLR = Platelet-to-Lymphocyte Ratio; PNI = Prognostic Nutritional Index; SII = Systemic Immune-Inflammation Index; SIRI = Systemic Inflammation Response Index.

**Table 2:** Univariable Cox regression analyses for overall survival and progression-free survival.

	Overall Survival			Progression-Free Survival		
Characteristic	HR	95% CI	*p*-Value	HR	95% CI	*p*-Value
Age, per 10 years	1.40	1.17 to 1.67	**0.0001**	1.23	1.06 to 1.44	**0.0065**
Sex			0.27			0.94
Male	—	—		—	—	
Female	0.78	0.50 to 1.21		1.01	0.68 to 1.51	
Comorbidity			0.14			0.16
No	—	—		—	—	
Yes	1.39	0.90 to 2.13		1.34	0.90 to 1.99	
Smoking			**0.033**			**0.032**
No	—	—		—	—	
Yes	1.65	1.05 to 2.57		1.58	1.05 to 2.39	
Prior brain tumor			0.44			0.53
No	—	—		—	—	
Yes	0.65	0.20 to 2.09		0.73	0.26 to 2.04	
ECOG performance status			0.16			0.17
0–1	—	—		—	—	
≥2	1.53	0.87 to 2.68		1.44	0.87 to 2.38	
Presenting symptom			0.13			**0.010**
Headache	—	—		—	—	
Seizure or syncope	2.12	1.24 to 3.61		1.96	1.21 to 3.19	
Visual disturbance	0.42	0.06 to 3.07		0.38	0.05 to 2.76	
Motor deficit	1.52	0.60 to 3.84		2.00	0.91 to 4.41	
Numbness	1.06	0.26 to 4.39		2.40	0.86 to 6.68	
Other	1.38	0.33 to 5.83		3.42	1.34 to 8.74	
Neurological deficit			0.20			0.46
No	—	—		—	—	
Yes	1.41	0.84 to 2.37		1.20	0.75 to 1.93	
Epileptic seizure			0.054			0.14
No	—	—		—	—	
Yes	1.74	1.02 to 2.98		1.46	0.90 to 2.36	
Histological diagnosis			0.66			0.52
Astrocytoma, IDH-mutant, grade 4	—	—		—	—	
Glioblastoma, IDH-wildtype	0.86	0.43 to 1.69		0.82	0.45 to 1.49	
IDH status			0.26			0.14
IDH-wildtype	—	—		—	—	
IDH-mutant	0.96	0.45 to 2.03		1.01	0.53 to 1.93	
Unknown	0.67	0.41 to 1.10		0.65	0.42 to 1.02	
Laterality			0.93			0.39
Left	—	—		—	—	
Right	1.02	0.66 to 1.57		1.19	0.80 to 1.77	
Tumor location			0.39			**0.030**
Frontal	—	—		—	—	
Parietal	0.63	0.34 to 1.15		0.58	0.33 to 1.01	
Temporal	0.85	0.47 to 1.54		1.00	0.59 to 1.69	
Occipital	0.50	0.15 to 1.63		0.39	0.12 to 1.31	
Multiple lobes	1.30	0.68 to 2.50		1.82	0.99 to 3.34	
Deep or midline	0.92	0.22 to 3.84		0.65	0.16 to 2.69	
Focality			0.069			0.32
Unifocal	—	—		—	—	
Multifocal	2.24	1.03 to 4.91		1.51	0.69 to 3.28	
Tumor size, per 10 mm	1.00	0.87 to 1.14	0.97	0.97	0.85 to 1.10	0.64
Ki-67, per 10%	0.99	0.87 to 1.12	0.85	1.00	0.90 to 1.12	0.95
Extent of resection			0.96			0.79
Biopsy	—	—		—	—	
Subtotal resection	0.86	0.25 to 2.94		0.99	0.34 to 2.85	
Gross total resection	0.92	0.28 to 2.98		0.85	0.31 to 2.38	
Hemoglobin, per SD	0.87	0.70 to 1.08	0.22	0.78	0.64 to 0.95	**0.017**
Albumin, per SD	0.88	0.70 to 1.10	0.26	0.93	0.75 to 1.15	0.50
C-reactive protein, per SD	1.23	0.98 to 1.55	0.10	1.16	0.94 to 1.44	0.20
Lactate dehydrogenase, per SD	0.91	0.71 to 1.17	0.47	1.01	0.84 to 1.22	0.90
Uric acid, per SD	1.07	0.84 to 1.37	0.56	1.06	0.85 to 1.31	0.63
White blood cells, per SD	1.10	0.91 to 1.34	0.34	0.99	0.82 to 1.18	0.88
Neutrophils, per SD	1.04	0.86 to 1.27	0.68	0.96	0.80 to 1.15	0.67
Lymphocytes, per SD	1.12	0.89 to 1.42	0.35	1.05	0.85 to 1.29	0.67
Monocytes, per SD	1.17	0.96 to 1.43	0.13	1.03	0.85 to 1.24	0.78
Platelets, per SD	0.84	0.66 to 1.08	0.17	0.91	0.74 to 1.14	0.42
NLR, per SD	1.03	0.83 to 1.28	0.76	0.97	0.80 to 1.17	0.72
dNLR, per SD	0.95	0.77 to 1.18	0.65	0.92	0.76 to 1.11	0.37
PLR, per SD	0.92	0.73 to 1.16	0.48	0.95	0.77 to 1.16	0.59
MLR, per SD	1.16	0.93 to 1.45	0.19	1.05	0.86 to 1.29	0.62
SII, per SD	0.99	0.80 to 1.22	0.89	0.94	0.77 to 1.14	0.50
SIRI, per SD	1.08	0.89 to 1.32	0.42	0.99	0.82 to 1.18	0.89
PIV, per SD	1.06	0.86 to 1.29	0.60	0.97	0.80 to 1.17	0.76
PNI, per SD	0.98	0.77 to 1.25	0.89	0.98	0.79 to 1.22	0.86
CAR, per SD	1.23	0.98 to 1.55	0.10	1.15	0.93 to 1.42	0.22
Adjuvant radiotherapy *			0.42			0.15
No	—	—		—	—	
Yes	1.57	0.49 to 5.04		2.14	0.66 to 6.93	
Adjuvant chemotherapy *			**0.0004**			**0.0029**
No	—	—		—	—	
Yes	0.04	0.01 to 0.16		0.08	0.02 to 0.29	
Neutropenia during chemotherapy *			0.63			0.67
No	—	—		—	—	
Yes	0.84	0.40 to 1.74		0.87	0.46 to 1.64	
Response to adjuvant therapy *			**0.0001**			**0.0002**
Complete response	—	—		—	—	
Residual disease under follow-up	2.53	1.08 to 5.96		1.77	0.84 to 3.73	
Not completed or progression during therapy	3.82	1.87 to 7.81		3.03	1.63 to 5.62	

Abbreviations: CI = Confidence Interval; HR = Hazard Ratio; CAR = C-Reactive Protein-to-Albumin Ratio; dNLR = Derived Neutrophil-to-Lymphocyte Ratio; ECOG = Eastern Cooperative Oncology Group; IDH = Isocitrate Dehydrogenase; MLR = Monocyte-to-Lymphocyte Ratio; NLR = Neutrophil-to-Lymphocyte Ratio; PIV = Pan-Immune-Inflammation Value; PLR = Platelet-to-Lymphocyte Ratio; PNI = Prognostic Nutritional Index; SD = Standard Deviation; SII = Systemic Immune-Inflammation Index; SIRI = Systemic Inflammation Response Index. * Treatment-related variables are subject to guarantee-time bias and were not included in multivariable models. Bold *p*-values indicate *p* < 0.05.

**Table 3:** Multivariable Cox regression models for overall survival and progression-free survival (stratified by center).

	Overall Survival (N = 120; 85 Events)			Progression-Free Survival (N = 119; 103 Events)		
Characteristic	HR	95% CI	*p*-Value	HR	95% CI	*p*-Value
SIRI						
Low (≤2.85)	—	—		—	—	
High (>2.85)	2.62	1.38 to 4.96	**0.0031**	1.24	0.68 to 2.25	0.48
Hemoglobin, per g/dL	0.95	0.81 to 1.10	0.49	0.84	0.73 to 0.96	**0.011**
Age, per 10 years	1.45	1.18 to 1.78	**0.0004**	1.25	1.04 to 1.50	**0.015**
Smoking						
No	—	—		—	—	
Yes	2.02	1.16 to 3.54	**0.013**	1.73	1.06 to 2.81	**0.029**
ECOG performance status						
0–1	—	—		—	—	
≥2	1.73	0.79 to 3.83	0.17	1.29	0.64 to 2.58	0.47
Gross total resection						
No GTR	—	—		—	—	
GTR	0.60	0.33 to 1.10	0.10	0.49	0.29 to 0.83	**0.0080**
IDH status						
IDH-wildtype	—	—		—	—	
IDH-mutant	0.70	0.24 to 2.09	0.53	0.79	0.31 to 2.04	0.63
Unknown	0.49	0.26 to 0.93	**0.029**	0.55	0.30 to 1.00	0.051

Abbreviations: CI = Confidence Interval; HR = Hazard Ratio; ECOG = Eastern Cooperative Oncology Group; GTR = Gross Total Resection; IDH = Isocitrate Dehydrogenase; SIRI = Systemic Inflammation Response Index. Multivariable Cox proportional-hazards models stratified by center. One patient with progression but unknown progression date was excluded from the progression-free survival analysis. Bold *p*-values indicate *p* < 0.05.

## Data Availability

The data presented in this study are available on request from the corresponding author due to patient privacy restrictions.

## Supplementary Materials

The following supporting information can be downloaded at: https://www.mdpi.com/article/10.3390/cancers18193192/s1, Figure S1: Sensitivity analysis of the SIRI cut-off value across percentiles; Figure S2: Comparison of survival prediction models; Table S1: Multivariable Cox model for overall survival in the IDH-wildtype subgroup; Table S2: Adjusted hazard ratios and *p* values for SIRI across alternative cut-off values; Table S3: Multivariable Cox regression models restricted to patients with known IDH status; Table S4: Cross-validated C-index values of the machine learning models.
