# Supplementary material for: Prognostic Value of Systemic Inflammation Markers and Clinical Factors in Grade 4 Diffuse Glioma: A Multicenter Retrospective Real-World Study
Source: Cancers (Basel). 2026 Oct 2;18(19):3192. doi: 10.3390/cancers18193192 (PMC13664342; doi:10.3390/cancers18193192)
Supplement: Supplementary file 1 [file cancers-18-03192-s001.zip › Supplementary_Materials.pdf]

Supplementary Materials

Prognostic Value of Systemic Inflammation Markers and Clinical Factors in Grade 4 Diffuse Glioma: A Multicenter Retrospective Real-World Study

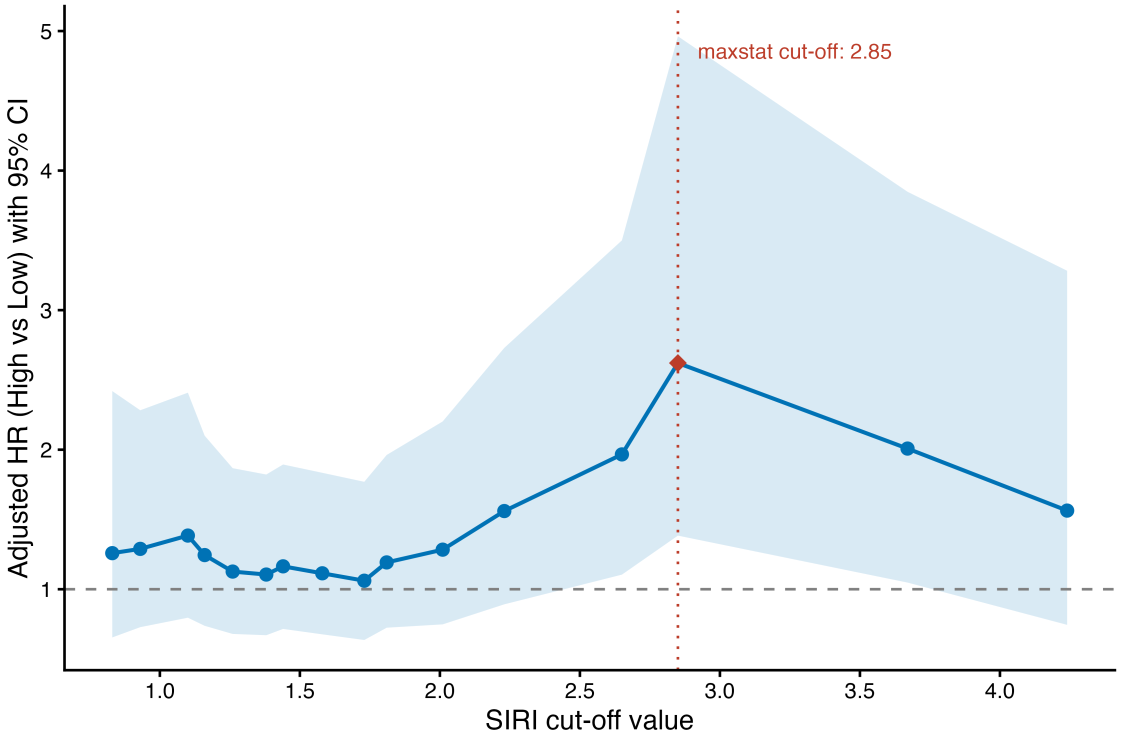

Figure S1. Sensitivity analysis of the SIRI cut-off value across percentiles.

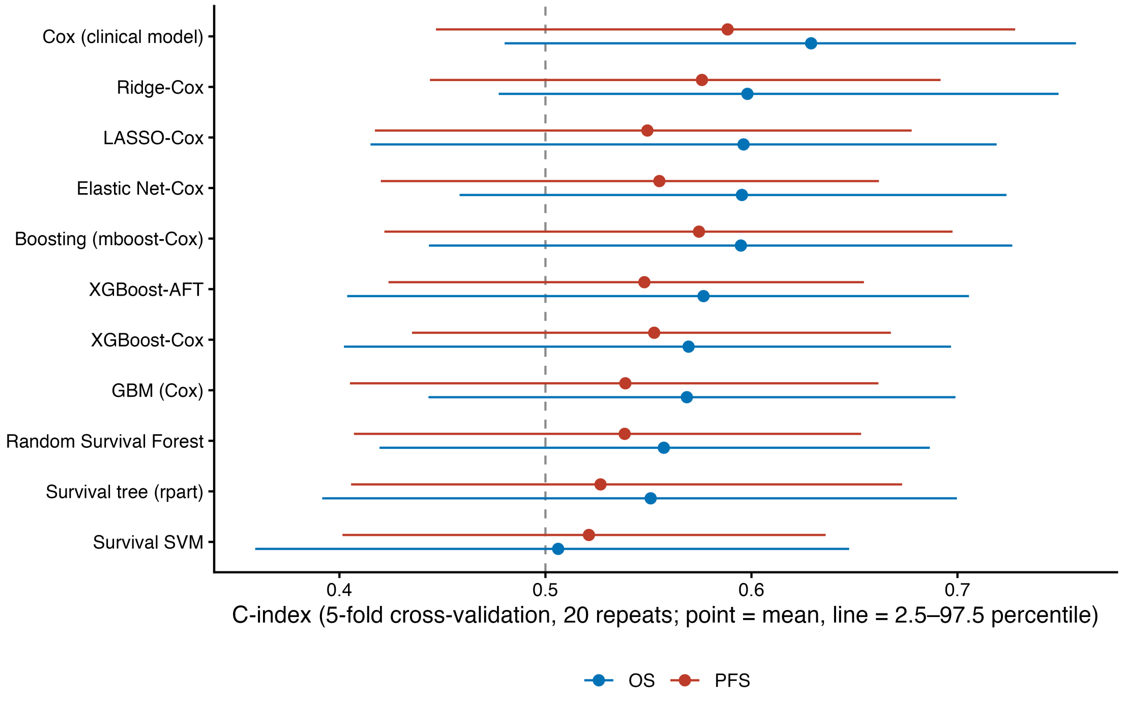

Figure S2. Comparison of survival prediction models.

Table S1. Multivariable Cox model for overall survival in the IDH-wildtype subgroup.

Variable	HR	95% CI	p-value
**SIRI**			
Low (≤2.85)	—	—	
High (>2.85)	3.06	1.27 to 7.39	**0.013**
**Hemoglobin, per g/dL**	0.95	0.74 to 1.22	0.70
**Age, per 10 years**	1.46	1.07 to 1.99	**0.018**
**Smoking**			
No	—	—	
Yes	2.45	1.17 to 5.15	**0.018**
**ECOG performance status**			
0–1	—	—	
≥2	3.11	0.92 to 10.5	0.068
**Gross total resection**			
No GTR	—	—	
GTR	0.29	0.12 to 0.72	**0.0079**

Abbreviations: CI = Confidence Interval, HR = Hazard Ratio

Overall survival, IDH-wildtype glioblastoma only (N=71; 51 deaths). Cox model stratified by center. HR=hazard ratio.

Bold *p*-values indicate $p < 0.05$.

Table S2. Adjusted hazard ratios and *p* values for SIRI across alternative cut-off values.

Cut-off value	Percentile	Patients above cut-off, n	Adjusted HR	95% CI	p value
0.83	15th	102	1.26	0.65–2.42	0.491
0.93	20th	96	1.29	0.73–2.28	0.383
1.10	25th	90	1.38	0.80–2.41	0.249
1.16	30th	84	1.24	0.74–2.10	0.412
1.26	35th	78	1.13	0.68–1.87	0.644
1.38	40th	72	1.11	0.67–1.82	0.695
1.44	45th	66	1.16	0.72–1.89	0.541
1.58	50th	60	1.11	0.68–1.83	0.671
1.73	55th	54	1.06	0.64–1.77	0.820
1.81	60th	48	1.19	0.72–1.96	0.489
2.01	65th	42	1.28	0.75–2.20	0.365
2.23	70th	36	1.56	0.89–2.73	0.119
2.65	75th	30	1.97	1.10–3.50	0.022
**2.85**	**maxstat cut-off**	**29**	**2.62**	**1.38–4.96**	**0.003**
3.67	80th	24	2.01	1.05–3.85	0.036
4.24	85th	18	1.56	0.74–3.28	0.238

Each row is a separate multivariable Cox model (SIRI dichotomized at the given value) with the covariates of the final model: hemoglobin, age, smoking, ECOG performance status, extent of resection, and IDH status, stratified by center. Percentiles refer to the SIRI distribution of the cohort (N = 120). HR = hazard ratio; CI = confidence interval.

Table S3. Multivariable Cox regression models restricted to patients with known IDH status.

	Overall survival (N=84; 59 events)			Progression-free survival (N=83; 71 events)		
**Characteristic**	**HR**	**95% CI**	**p-value**	**HR**	**95% CI**	**p-value**
**SIRI**						
Low (≤2.85)	—	—		—	—	
High (>2.85)	2.02	0.87 to 4.66	0.10	0.65	0.29 to 1.47	0.30
**Hemoglobin, per g/dL**	0.95	0.77 to 1.16	0.58	0.87	0.73 to 1.03	0.10
**Age, per 10 years**	1.36	1.03 to 1.79	**0.030**	1.12	0.88 to 1.42	0.37
**Smoking**						
No	—	—		—	—	
Yes	2.58	1.26 to 5.28	**0.0093**	2.04	1.04 to 4.00	**0.039**
**ECOG performance status**						
0–1	—	—		—	—	
≥2	2.58	0.95 to 7.04	0.064	2.19	0.84 to 5.66	0.11
**Gross total resection**						
No GTR	—	—		—	—	
GTR	0.34	0.15 to 0.75	**0.0080**	0.30	0.14 to 0.62	**0.0012**
**IDH status**						
IDH-wildtype	—	—		—	—	
IDH-mutant	0.37	0.11 to 1.31	0.12	0.45	0.14 to 1.42	0.17

Abbreviations: CI = Confidence Interval, HR = Hazard Ratio

Multivariable Cox models stratified by center, restricted to patients with known IDH status. HR=hazard ratio.

Bold *p*-values indicate $p < 0.05$.

Table S4. Cross-validated C-index values of the machine learning models.

(a) Overall survival

**Model**	**Mean C-index**	**SD**	**2.5th–97.5th percentile**
Cox (clinical model)	0.629	0.072	0.480–0.758
Ridge-Cox	0.598	0.076	0.477–0.749
LASSO-Cox	0.596	0.075	0.415–0.719
Elastic Net-Cox	0.595	0.079	0.458–0.724
Boosting (mboost-Cox)	0.595	0.072	0.443–0.727
XGBoost-AFT	0.577	0.077	0.404–0.706
XGBoost-Cox	0.569	0.074	0.402–0.697
GBM (Cox)	0.569	0.065	0.443–0.699
Random Survival Forest	0.557	0.077	0.419–0.687
Survival tree (rpart)	0.551	0.083	0.392–0.700
Survival SVM	0.506	0.077	0.359–0.647

5-fold cross-validation repeated 20 times (100 test folds); C-index = Harrell's concordance index.

(b) Progression-free survival

**Model**	**Mean C-index**	**SD**	**2.5th–97.5th percentile**
Cox (clinical model)	0.588	0.075	0.447–0.728
Ridge-Cox	0.576	0.065	0.444–0.692
Boosting (mboost-Cox)	0.575	0.069	0.422–0.698
Elastic Net-Cox	0.555	0.065	0.420–0.662
XGBoost-Cox	0.553	0.064	0.435–0.668
LASSO-Cox	0.549	0.068	0.417–0.678
XGBoost-AFT	0.548	0.067	0.424–0.655
GBM (Cox)	0.539	0.070	0.405–0.662
Random Survival Forest	0.538	0.070	0.407–0.653
Survival tree (rpart)	0.527	0.069	0.406–0.673
Survival SVM	0.521	0.067	0.401–0.636

5-fold cross-validation repeated 20 times (100 test folds); C-index = Harrell's concordance index.
